# Elevated tumor necrosis factor alpha and vascular endothelial growth factor in intermediate age-related macular degeneration and geographic atrophy

**DOI:** 10.3389/fopht.2024.1356957

**Published:** 2024-03-26

**Authors:** Vivian Rajeswaren, Brandie D. Wagner, Jennifer L. Patnaik, Naresh Mandava, Marc T. Mathias, Niranjan Manoharan, Talisa E. de Carlo Forest, Ramya Gnanaraj, Anne M. Lynch, Alan G. Palestine, the University of Colorado Retina Research Group

**Affiliations:** ^1^ Department of Ophthalmology, University of Colorado School of Medicine, Aurora, CO, United States; ^2^ Colorado School of Public Health, University of Colorado School of Medicine, Aurora, CO, United States

**Keywords:** age-related macular degeneration, tumor necrosis factor alpha, vascular endothelial growth factor, intermediate age related macular degeneration, geographic atrophy

## Abstract

**Introduction:**

Tumor necrosis factor alpha (TNF-α) is an inflammatory cytokine implicated in pathological changes to the retinal pigment epithelium that are similar to changes in geographic atrophy (GA), an advanced form of age related macular degeneration (AMD). TNF-α also modulates expression of other cytokines including vascular endothelial growth factor (VEGF), leading to choroidal atrophy in models of AMD. The purpose of this study was to investigate systemic TNF-α and VEGF in patients with GA and intermediate AMD (iAMD) compared to controls without AMD.

**Methods:**

We examined plasma levels of TNF-α and VEGF in patients with GA, iAMD, and controls without AMD from the University of Colorado AMD registry (2014 to 2021). Cases and controls were characterized by multimodal imaging. TNF-α and VEGF were measured via multiplex immunoassay and data were analyzed using a non-parametric rank based linear regression model fit to plasma biomarkers.

**Results:**

There were 97 GA, 199 iAMD patients and 139 controls. TNF-α was significantly increased in GA (Median:9.9pg/ml, IQR:7.3-11.8) compared to iAMD (Median:7.4, IQR:5.3-9.1) and in both GA and iAMD compared to controls (Median:6.4, IQR:5.3-7.8), p<0.01 for all comparisons. VEGF was significantly increased in iAMD (Median:8.9, IQR:4.8-14.3) compared to controls (Median:7.7, IQR:4.6-11.1), p<0.01. There was a significant positive correlation between TNF-α and VEGF in GA (0.46, p<0.01), and iAMD (0.20, p=0.01) with no significant interaction between TNF-α and VEGF in any group.

**Discussion:**

These findings suggest TNF-α and VEGF may contribute to systemic inflammatory processes associated with iAMD and GA. TNF-α and VEGF may function as systemic biomarkers for disease development.

## 1 Introduction

Age-related macular degeneration (AMD) is a degenerative disease of the central retina, which is crucial for high acuity vision (1). It is the third most common cause of visual impairment globally, and is predicted to affect 288 million individuals worldwide by 2040 (1, 2). The hallmark of AMD is the presence of drusen between the retinal pigment epithelium (RPE) and Bruch’s membrane (3, 4). Early AMD is characterized by extracellular deposition of drusen and intermediate AMD (iAMD) by retinal pigmentary alterations in addition to drusen deposition (3, 4). Advanced AMD is classified into two forms, neovascular AMD (nvAMD) and geographic atrophy (GA) (3, 5). GA is defined by atrophic lesions due to the progressive loss of photoreceptors, RPE, and choriocapillaris, eventually causing permanent vision loss (5). Patients with iAMD are at risk for progression to advanced AMD (6, 7). GA affects an estimated 1 million individuals in the United States, resulting in increased economic burden (8) and poor health related quality of life (9).

Chronic local and systemic inflammation play a role in the pathogenesis and progression of AMD (10–13). Recent studies from our research group suggest that systemic tumor necrosis factor alpha (TNF-α) may play a role in iAMD (14, 15). TNF-α is an inflammatory cytokine mediating cellular differentiation, proliferation, and survival (16, 17). In cellular models of GA, expression of TNF-α is thought to underlie RPE cell death in response to chronic oxidative stress (18, 19). Furthermore, RPE cells chronically exposed to TNF-α undergo pathological changes to RPE morphology and function similar to those found in GA (20). TNF-α also moderates the expression of several inflammatory cytokines including vascular endothelial growth factor (VEGF) (21, 22). Terasaki and colleagues found that subretinal injection of TNF-α led to decreased expression of VEGF in RPE cells compared to control cells, which was inhibited by co-injection of an antibody to TNF-α (23). In addition, choroidal thickness post-injection was decreased compared to controls (23). The absence of apoptotic cells on terminal deoxynucleotidyl transferase dUTP nick end labeling (TUNEL) points away from TNF-α induced apoptosis of choroidal cells and suggests a role for TNF-α mediated decrease in VEGF in atrophy of the choriocapillaris (23). Therefore, systemic TNF-α and VEGF may be important in the pathogenesis of iAMD and GA and may function as biomarkers for the onset and targeted treatment of this vision threatening disease.

Previous research on inflammatory cytokines in AMD has focused on cytokines locally in the intraocular fluid (24–28) or in cellular AMD models (20, 27, 29–31) However, to our knowledge there is minimal research examining systemic plasma levels of both TNF-α and VEGF and the potential interaction of these two cytokines in GA and the intermediate form of AMD. Thus, the purpose of this study was to build on our prior findings in iAMD and examine systemic concentrations of TNF-α and VEGF in patients with GA and iAMD compared to controls with no AMD.

## 2 Materials and methods

### 2.1 Study design and population

This study was conducted using records and samples from patients with GA, iAMD and controls with no AMD from an AMD research registry developed by the University of Colorado, Department of Ophthalmology (32, 33). The registry is approved by the Colorado Multiple Institutional Review Board (IRB) and is in accordance with the Declaration of Helsinki. Patients attending the retina clinics at the Sue Anschutz-Rodgers Eye are invited to participate in the registry. Written informed consent for collection of serum and plasma for the study of biomarkers, review of relevant medical history, and inspection of multimodal imaging (color fundus photography, autofluorescence, and ophthalmic coherence tomography) is obtained from all patients who agree to participate in the registry (14, 15).

### 2.2 Inclusion and exclusion criteria for the University of Colorado AMD registry

Informed consent, inclusion criteria and exclusion criteria are explained in detail elsewhere (34–36). Briefly, the inclusion criteria for the AMD registry include patient age between 55 and 99 years, capacity for informed consent, and unilateral or bilateral AMD. Exclusion criteria include proliferative and nonproliferative diabetic retinopathy, cystoid macula edema, diabetic macula edema, central serous retinopathy, active inflammatory ocular disease, branch and central retinal vein occlusion, macula off retinal detachment, full-thickness macular hole, pattern or occult macular dystrophy, ocular melanoma, drusen not caused by iAMD or GA, macular telangiectasia, corneal transplant, prior or current treatment with anti-VEGF injections, pan retinal photocoagulation, current systemic treatment for cancer, serious mental health issues or advanced dementia and terminal illness. Controls are patients who had cataract surgery one month or greater prior to enrollment without evidence of retinal disease on multimodal imaging. Registry data is entered into REDCap, a secure web-based IRB-approved database.

### 2.3 Specific inclusion criteria for this study

For this current study, the analytic data set was restricted to participants from the registry with GA, iAMD and controls recruited between August 2014 and June 2021. Patients with nvAMD were excluded from this analytic dataset.

### 2.4 Image review

Two vitreoretinal specialists (authors MM and NM) conducted a multimodal image review to confirm the AMD phenotype. Discrepancies were resolved by a third vitreoretinal specialist (author TDCF). The Beckman Initiative for Macular Research Classification Committee Image criteria was used to classify AMD phenotype (37) and GA was additionally characterized into complete RPE and outer retinal atrophy according to the Classification of Atrophy Meeting (CAM) (38, 39). Criteria were as follows: (a) a region of choroidal hyper transmission larger than or equal to 250 μm in diameter; (b) a region of attenuation or disruption to the RPE larger than or equal to 250 μm in diameter; and (c) evidence of overlying photoreceptor degeneration. All criteria were defined in absence of a tear in the RPE (38, 39).

### 2.5 Blood sample collection and processing

A cooled (4° C) centrifuge set at 3000 rpm was used to spin Ethylenediaminetetraacetic acid (EDTA) plasma samples for 10 minutes. Plasma aliquots were immediately stored in a −80° C freezer and transferred to the laboratory for measurement of biomarkers (40).

### 2.6 Measurement of plasma biomarkers

Levels of the plasma biomarkers TNF-α and VEGF were measured at the Children’s Hospital Colorado by the Clinical Translational Research Core laboratory. Analyte-specific antibodies were analyzed using a magnetic bead-based multiplex assay (R&D systems) on the Luminex FlexMap, a dual-laser suspension array platform. Duplicate analysis was conducted on all samples with a threshold coefficient of variance less than 15% (41).

### 2.7 Statistical analysis

A two-sample *t*-test for continuous variables and the χ^2^ test or Fisher’s exact test for categorical variables were used to compare patient characteristics across groups. Covariates included age, family history of AMD, smoking, body mass index (BMI), race, Hispanic ethnicity, and a history of treated hypertension, kidney disease, peripheral vascular disease, cardiac disease, and diabetes. Spearman’s rank correlation coefficient was measured between TNF-α, VEGF and age across AMD groups. A regression model using a rank transformation was fit to TNF-α and VEGF with the following covariates, AMD classification, age, race and assay run date. A multinomial regression model was run with AMD group as a three-level categorical outcome and VEGF, TNF-α, assay run date and age as independent variables. A sensitivity analysis was performed excluding all non-White subjects and a regression model with AMD group as a categorical outcome was repeated with VEGF, TNF-α, assay run date and age as independent variables. All analyses were performed using SAS version 9.4 (SAS Institute Inc., Cary, NC, USA).

## 3 Results

### 3.1 Demographic and clinical characteristics

**Table 1** illustrates select demographic characteristics across GA, iAMD and control patients. The greatest proportion of females (66%) was in the control group, followed by the iAMD group (61%) and GA groups (55%). Sex distribution was not significant across groups. Mean patient age was highest in the GA group (82.3, SD: 7.0), followed by the iAMD (76.4, SD: 6.9) and control (74.2, SD: 4.7) groups, p < 0.01. Race significantly differed across groups, (p < 0.01), with the lowest percentage of White race (86%) and highest percentage of Black/African American race (8%) in the control group. In addition, a reported family history of AMD significantly differed across groups, (p < 0.01), with the highest levels in the iAMD cohort. Prevalence of treated hypertension was significantly different across groups with the greatest prevalence in GA patients at 73%, with 54% in controls and 53% in iAMD patients, p < 0.01.

**Table 1:** Clinical characteristics across AMD Groups.

	GA (n = 97)	iAMD (n = 199)	Controls (n = 139)	p-value*
Sex, Female	53 (55%)	122 (61%)	92 (66%)	0.20
Race				<0.01
White	94 (97%)	192 (96%)	119 (86%)	
Black or African American	0	2 (1%)	11 (8%)	
Native American/Alaska Native	0	1 (0.5%)	0	
Asian	1 (1%)	1 (0.5%)	1 (1%)	
Other	1 (1%)	3 (2%)	5 (3%)	
Uncertain	1 (1%)	0	3 (2%)	
Hispanic Ethnicity	5 (5%)	4 (2%)	6 (4%)	0.12^†^
Family History of AMD				<0.01^†^
None	56 (58%)	101 (51%)	106 (77%)	
Yes	28 (29%)	72 (36%)	26 (19%)	
Uncertain	13 (13%)	26 (13%)	6 (4%)	
Age, Mean (SD)	82.3 (7.0)	76.4 (6.9)	74.2 (4.7)	<0.01
Body Mass Index, Mean (SD)	26.7 (5.0) n = 84	27.0 (5.3) n = 194	27.3 (5.6) n = 136	0.65
Smoking				0.40^†^
Never	39 (40%)	92 (46%)	72 (52%)	
Current	2 (2%)	6 (3%)	2 (1%)	
Former	56 (58%)	101 (51%)	64 (46%)	
History of:				
Treated hypertension	71 (73%)	106 (53%)	75 (54%)	<0.01
Kidney disease	17 (18%)	27 (14%)	12 (9%)	0.13
Peripheral vascular disease	10 (10%)	32 (16%)	21 (15%)	0.07
Cardiac disease	41 (42%)	67 (34%)	42 (30%)	0.15
Diabetes	15 (15%)	29 (15%)	18 (13%)	0.85

*p-values obtained from Chi-square for categorical variables and ANOVA for continuous variables.

^†^p-value calculated from Fisher’s exact test.

### 3.2 Systemic levels of TNF-α and VEGF

**Figure 1:**
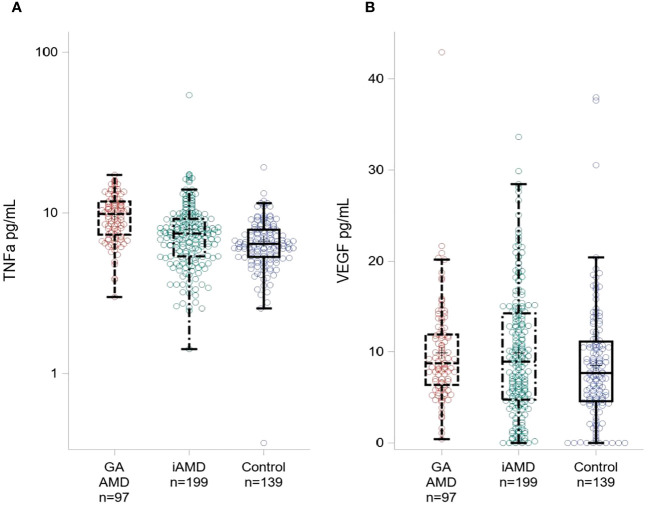
Plasma Levels of TNF-α and VEGF across AMD Groups. Boxplot showing TNF-α levels **(A)** and VEGF levels **(B)** for GA, iAMD and controls. The box extends to the 25^th^ and 75^th^ percentiles, the line represents the median value. Individual colored circles illustrate raw values.

In **Table 2** and **Figure 1**, we show systemic levels of TNF-α and VEGF in cases of GA, iAMD and controls. Levels of TNF-α were significantly elevated in GA compared to iAMD and controls, p < 0.01 for both comparisons. There was also a significant elevation in levels of TNF-α in the iAMD group compared to controls, p = 0.01. VEGF levels were significantly higher in iAMD compared to control patients, p < 0.01. There were no significant differences in levels of VEGF between the other groups. Results were also adjusted for the possible confounding variables of age, race and batch effects. P-values for TNF-α and VEGF were unchanged after a regression analysis with age, race and assay run date as covariates.

**Table 2:** TNF-α and VEGF levels in GA, iAMD and Controls.

Median (IQR)	GA (n = 97)	iAMD (n = 199)	Controls (n = 139)	GA vs iAMD p-value*	GA vs Control p-value*	iAMD vs Control p-value*
TNF-α (pg/mL)	9.9 (7.3 – 11.8)	7.4 (5.3 – 9.1)	6.4 (5.3 – 7.8)	<0.01	<0.01	0.01
VEGF (pg/mL)	8.8 (6.4 – 11.9)	8.9 (4.8 – 14.3)	7.7 (4.6 – 11.1)	0.54	0.12	<0.01

*p-values are estimated from a model that includes age and assay run date as covariates

### 3.3 Correlation between TNF-α, VEGF and age across AMD groups

**Figure 2:**
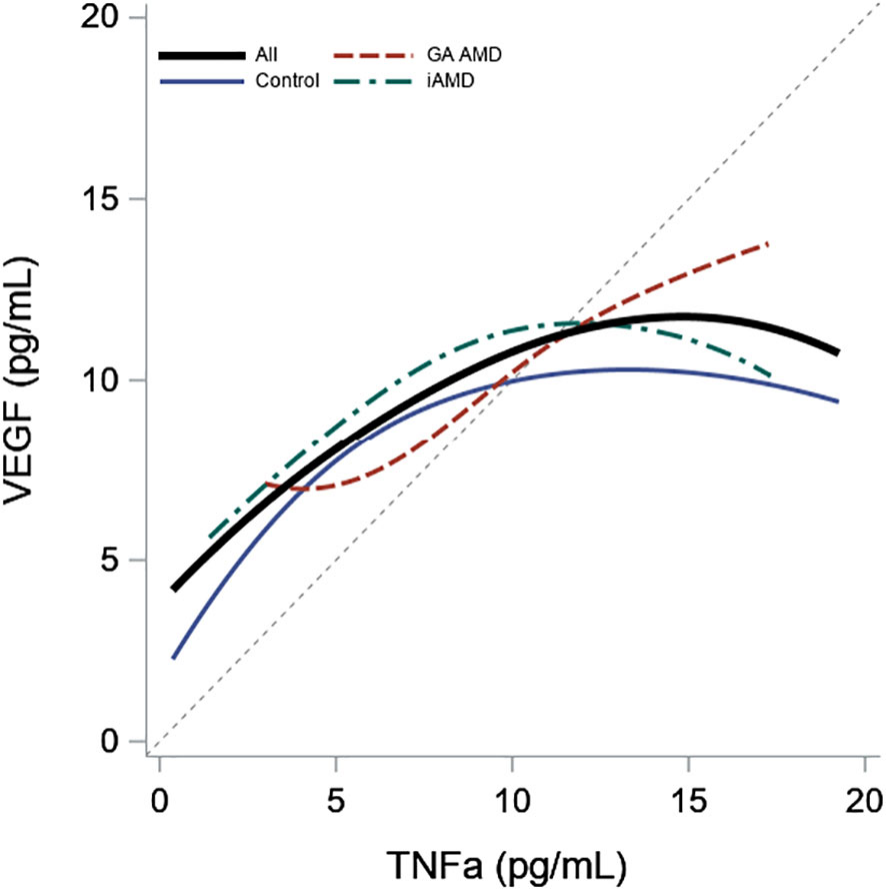
Association of Plasma Levels of TNF-α and VEGF by AMD Group. Scatterplot smoother illustrating the correlation between VEGF and TNF**-**α levels for all subjects and within each AMD group. A diagonal reference line is included for reference.

In **Table 3** and **Figure 2**, TNF-α and age were significantly positively correlated in all subjects (0.27, p < 0.01) and in the control group (0.23, p = 0.01). Correlations between VEGF and age were not significant in any of the groups. We also found a significant positive correlation between TNF-α and VEGF between all subjects (0.23, p < 0.01). When evaluated by group, the correlations were significant in GA and iAMD with the largest association in GA (0.46, p < 0.01), followed by iAMD (0.20, p = 0.01). The correlation in controls did not reach the level of significance.

**Table 3:** Spearman correlation coefficients between TNF-α, VEGF and age across AMD Groups.

Spearman Correlation (p)				
Variables	All subjects N = 435	GA N= 97	iAMD n = 199	Controls N = 139
TNF-α & age	0.27 (<0.01)	0.16 (0.12)	0.08 (0.29)	0.23 (0.01)
VEGF & age	0.07 (0.16)	-0.06 (0.55)	0.06 (0.43)	0.02 (0.82)
TNF-α & VEGF	0.23 (<0.01)	0.46 (<0.01)	0.20 (0.01)	0.11 (0.21)

### 3.4 Interactions between TNF-α and VEGF across AMD groups

A multinomial regression model with AMD group as a categorical outcome was run with VEGF, TNF-α, assay run date and age as independent variables. There was no significant interaction between VEGF and TNF-α (p = 0.81). Results from the model excluding the interaction show that TNF-α significantly distinguished controls from iAMD and GA after adjusting for VEGF, age and assay run date with an OR of 1.2 (95% CI: 1.1, 1.4) for GA compared to controls and 1.2 (1.1, 1.3) for iAMD compared to controls. There was no significant difference between GA and iAMD (p = 0.09). VEGF levels only significantly discriminated between iAMD and controls with an OR of 1.1 (1.01, 1.1). Results were unchanged when the regression model was run with a sensitivity analysis excluding all non-White subjects.

## 4 Discussion

### 4.1 Key study findings

We demonstrated significantly elevated median levels of TNF-α in GA compared to both iAMD and controls and between iAMD and control subjects. We also found significantly higher median VEGF levels in the iAMD group in comparison to controls. Median VEGF levels in the GA group were similar to the iAMD group but were not significantly higher than the control group. TNF-α and VEGF were significantly positively correlated in GA and iAMD with no significant interaction between TNF-α and VEGF in any group.

### 4.2 Comparison to prior research

TNF-α is a cytokine with a key role in regulating the inflammatory response (22). TNF-α is largely secreted by T helper 1 (Th1) cells and macrophages, inducing signal transduction pathways leading to cellular differentiation, expansion, and survival (16). However, excess production of TNF-α is linked to chronic inflammation and is implicated in the pathogenesis of several inflammatory and autoimmune diseases (16, 22). Plasma levels of TNF-α are elevated in rheumatoid arthritis (RA) (42), Henoch-Schonlein purpura (HSP) (43), immunoglobulin A vasculitis (IgAV) (44), chronic obstructive pulmonary disease (COPD) (45), psoriasis (PS) (46), and ankylosing spondylitis (AS) (47) and are associated with the severity of disease (42–47). Proposed mechanisms are the expression, recruitment and activation of inflammatory mediators and destructive enzymes through TNF-α signaling pathways which leads to localized pathological tissue damage (16, 22).

Plasma TNF-α in GA was elevated compared to iAMD and control subjects. Our results are in line with the significantly elevated plasma TNF receptor 2 levels (sTNFRII), a soluble receptor for TNF-α, in GA compared to healthy controls reported by Krogh Nielsen and colleagues (48). Klein et al. did not find a significant association between plasma levels of TNF-α and advanced AMD (49). Our results were not consistent with these findings, which may be due our study cohort examining GA instead of an advanced AMD group including both GA and nvAMD.

Plasma levels of TNF-α were elevated in iAMD in comparison to controls. These findings are aligned with our prior research showing elevated TNF-α in iAMD was associated with advanced AMD (14, 15). Our results differed from a study by Litwińska et al. which reported decreased levels of TNF-a in the plasma of dry AMD subjects (50). This may be due to the use of multimodal imaging in our study to characterize our patients into iAMD and GA compared to a single dry AMD group and the differing racial composition in our study populations. A study by Liisborg et al. described a significant difference in plasma TNF-α across iAMD, nvAMD, and myeloproliferative neoplasms with drusen (MPNd) and without drusen (MPNn) (51). It is difficult to compare the results of our study with these findings as we analyzed TNF-α levels in iAMD relative to levels in GA and controls with no inflammatory disease. Spindler and colleagues (52) showed no difference in plasma TNF-α between iAMD and controls. The difference in our results may be due to the larger sample sizes in our study and the disparate AMD classification system used to characterize our iAMD subjects.

Studies analyzing ocular fluid samples did not find a significant difference in levels of TNF-α between iAMD or GA patients compared to controls (24, 25). Our findings are in contrast to these results which may be due to the larger sample sizes in our study and different ethnic background of our study population. Spindler et al. demonstrated decreased TNF-a in the aqueous humor of dry AMD patients compared to healthy controls (52). Our findings are not consistent with these results, which may be a consequence of classifying our dry AMD subjects into iAMD and GA.

Oxidative stress induced AMD cell models showed a significant increase in the production of TNF-α (29, 53–55). Moreover, application of an anti-inflammatory agent reduced vitreous TNF-α expression in murine models of AMD (27). Touhami et al. demonstrated that RPE cell cultures chronically exposed to TNF-α displayed dose-dependent decreases in cell density, loss of barrier function, and morphological changes (20) similar to those observed in models of iAMD (30) and GA (31). Inflammatory eye diseases such as AMD lead to dysfunction of the blood-retinal-barrier (BRB) (56). Studies have shown that local retinal inflammation upregulates leukocyte chemoattractant molecules, promoting migration of peripheral blood leukocytes into the retina through permeable vessels of the choroid (56). We hypothesize that through a similar mechanism, the RPE is exposed to plasma cytokines through the damaged BRB which influence the pathogenesis of AMD. Thus, the elevation in plasma TNF-α may underly pathological damage to the retina as the disease progresses from iAMD to GA. The role of TNF-α as a potential therapeutic focus deserves investigation.

VEGF is a pleotropic cytokine mainly produced by endothelial cells, macrophages and activated T cells (57). VEGF has proangiogenic functions stimulating the growth and permeability of blood vessels (58) and operates as a proinflammatory cytokine by increasing permeability and adhesion of endothelial cells and acting as a chemoattractant for monocytes (57). VEGF also has a role in downregulating inflammation (59) and inhibiting apoptosis (60, 61). Circulating VEGF levels are elevated in several autoimmune diseases including systemic lupus erythematosus (SLE), RA, AS, inflammatory bowel disease (IBD), psoriatic arthritis (PsA), systemic sclerosis (SSc) (62), Bechet’s disease and multiple sclerosis (MS) (63) with a positive association to disease activity and severity (62, 63). Although the function of VEGF in these pathologies is not fully established, it has been suggested that angiogenesis, proinflammation, and anti-apoptosis all play a role in chronic inflammatory disease (64, 65).

In our study, we demonstrated elevated plasma VEGF in iAMD patients when compared to the control group. In a study by Ioanna et al. there were was no significant difference in plasma VEGF levels in dry AMD patients compared to controls (66). Possible reasons for the difference in our results include the classification of patients into iAMD and GA in our cohort and a larger sample size in our study. Sharma et al. described elevated levels of VEGF in AMD compared to controls with no difference in VEGF levels between dry and wet AMD (67). It is difficult to compare our results to these findings as our study analyzed VEGF levels in iAMD and GA instead of a single AMD group and there were no subjects with nvAMD in our study. Liisborg et al. reported a significant difference in VEGF across iAMD, nvAMD, MPNd and MPNn (51). Our findings are not comparable with these results as our study analyzed VEGF levels in iAMD relative to GA and control groups.

In the aqueous humor, measurement of VEGF levels in iAMD have yielded variable results. Muether et al. (26) showed elevated VEGF in iAMD patients compared to controls, which is consistent with our findings. However, our results are disparate from a recent report of no difference in VEGF levels in the aqueous humor between iAMD and controls (68). This may be due to the much larger sample sizes with sufficient power to detect a significant difference in cytokine levels in our study.

Cellular models of AMD have shown a significant increase in the production of VEGF in response to oxidative stress (28, 29, 69, 70) and a role for VEGF in the maintenance of the choriocapillaris (71, 72). Evidence suggests VEGF exhibits neuroprotective effects in the central nervous system (73, 74). Exposure to hypoxic-ischemic injury induced expression of VEGF and its receptors (60, 61) and conferred apoptotic resistance to hippocampal (61) and cerebellar granular neurons that diminished with application of VEGF neutralizing antibodies (60). Moreover, multiple models of murine optic nerve injury showed upregulation of VEGF, inhibition of apoptosis and increased survival of retinal ganglion cells (75). VEGF mediated neuroprotection described by other authors (60, 61, 73–75) may indicate a protective role for VEGF in iAMD. Given the role of VEGF in nvAMD (76), further study is warranted to investigate differences in systemic VEGF in GA and nvAMD.

We found a significant positive statistical correlation between TNF-α and VEGF in GA and iAMD. However, we did not find a significant statistical interaction between levels of TNF-α and VEGF for GA and iAMD compared to controls or in GA compared to iAMD. Our results are in contrast to studies showing that polarized RPE cells exposed to TNF-α showed downregulation of VEGF expression and atrophy of the RPE and choroid (23). Median VEGF levels in our study were similarly elevated in GA and iAMD but VEGF levels only significantly differed between iAMD and controls. This is likely due to the much smaller sample size in the GA group. The positive correlation between TNF-α and VEGF indicates a linkage between cytokine levels in iAMD and GA. However, the smaller sample size in our GA group may have been insufficient to detect a significant difference in median levels of VEGF between GA and the other groups or an interaction between TNF-α and VEGF across AMD groups. It will be important to investigate a potential interaction between TNF-α and VEGF in GA in future studies.

### 4.3 Limitations

This study is limited by the single time point measurement of cytokines and a relatively small sample size, particularly in the GA cohort. Information on the duration of disease before blood sample collection for all patients in our cohort is not available due to lack of access to historical patient records. Measurement of disease duration before plasma collection and longitudinal values of plasma cytokines in a larger patient cohort will be important in future studies as cytokine profiles may vary with the duration of AMD. In addition, we are unable to assess our patient population for the presence of pseudoexfoliation glaucoma (PE) due to lack of access to pre-operative records for some individuals with prior cataract surgery. Elevated plasma levels of TNF-α have been described in patients with PE (77), which may have influenced levels of TNF-α in our study. There is also evidence that fundus autofluorescence (FAF) pattern may impact GA progression (78). In future studies it will be important to assess FAF pattern for GA to determine if there is an impact on disease progression. Lastly, evidence from clinical trials suggests that multimodal imaging, including OCT, FAF, and infrared imaging is the best method for diagnosing GA (79–83). Our study is limited by a single method of imaging to diagnose GA.

### 4.4 Conclusion

To conclude, these results suggest TNF-α and VEGF may play a role in chronic inflammation in iAMD and GA. Further evaluation of the interaction between TNF and VEGF will be important to elucidate the mechanisms underlying these systemic pathways and understand the role of systemic biomarkers for earlier diagnosis and specific therapies for AMD.

## Data availability statement

The data analyzed in this study is subject to the following licenses/restrictions: The data that support the findings of this study are not openly available due to reasons of participant privacy and are available from the authors upon reasonable request. Requests to access these datasets should be directed to anne.lynch@cuanschutz.edu.

## Ethics statement

The studies involving humans were approved by Colorado Multiple Institutional Review Board. The studies were conducted in accordance with the local legislation and institutional requirements. The participants provided their written informed consent to participate in this study.

## Author contributions

VR: Writing – original draft, Writing – review & editing. BW: Formal analysis, Writing – review & editing. JP: Methodology, Writing – review & editing. NaM: Conceptualization, Funding acquisition, Methodology, Writing – review & editing. MM: Methodology, Writing – review & editing. NiM: Methodology, Writing – review & editing. TD: Methodology, Writing – review & editing. RG: Data curation, Writing – review & editing. AL: Conceptualization, Funding acquisition, Writing – review & editing. AP: Conceptualization, Writing – review & editing.

## Group members of University of Colorado Retina Research Group

Melanie Akau OD, Rebecca Baldermann BA, Karen L. Christopher MD, Richard Davidson MD, Talisa E. De Carlo Forest MD, C. Rob Graef OD, Ramya Gnanaraj MD, Anne M. Lynch MB, BCH, BAO, MSPH, Naresh Mandava MD, Niranjan Manoharan MD, Marc T. Mathias MD, Scott N. Oliver MD, Jeffery L. Olson MD, Alan G. Palestine MD, Jennifer L. Patnaik PhD, MHS, Jesse M. Smith MD, Brandie D. Wagner PhD.
